# COgnitive behavioural therapy versus standardised medical care for adults with Dissociative non-Epileptic Seizures (CODES): statistical and economic analysis plan for a randomised controlled trial

**DOI:** 10.1186/s13063-017-2006-4

**Published:** 2017-06-06

**Authors:** Emily J. Robinson, Laura H. Goldstein, Paul McCrone, Iain Perdue, Trudie Chalder, John D. C. Mellers, Mark P. Richardson, Joanna Murray, Markus Reuber, Nick Medford, Jon Stone, Alan Carson, Sabine Landau

**Affiliations:** 1 Department of Biostatistics & Health Informatics, Institute of Psychiatry, Psychology and Neuroscience, King’s College London, Denmark Hill Campus, London, SE5 8AF UK; 2 Department of Psychology, Institute of Psychiatry, Psychology and Neuroscience, King’s College London, Denmark Hill Campus, London, SE5 8AF UK; 3 Department of Health Service and Population Research, Institute of Psychiatry, Psychology and Neuroscience, King’s College London, Denmark Hill Campus, London, SE5 8AF UK; 4 Department of Psychological Medicine, Institute of Psychiatry, Psychology and Neuroscience, King’s College London, Denmark Hill Campus, London, SE5 8AF UK; 5 Department of Neuropsychiatry, Maudsley Hospital, South London and Maudsley NHS Foundation Trust, Denmark Hill Campus, London, SE5 8AZ UK; 6 Department of Basic and Clinical Neuroscience, Institute of Psychiatry, Psychology and Neuroscience, King’s College London, Denmark Hill Campus, London, SE5 8AF UK; 7 Academic Neurology Unit, Royal Hallamshire Hospital, University of Sheffield, Glossop Road, Sheffield, S10 2JF UK; 8 The Lishman Unit, South London and Maudsley NHS Foundation Trust, Denmark Hill, London, SE5 8AZ UK; 9 Department of Clinical Neurosciences, Western General Hospital, University of Edinburgh, Edinburgh, EX4 2XU UK; 10 Centre for Clinical Brain Sciences, Western General Hospital, University of Edinburgh, Edinburgh, EX4 2XU UK

**Keywords:** Statistical analysis plan, CODES trial, Non-epileptic seizures, Dissociative seizures, Cognitive behavioural therapy, Randomised controlled trial

## Abstract

**Background:**

Dissociative seizures (DSs), also called *psychogenic non-epileptic seizures*, are a distressing and disabling problem for many patients in neurological settings with high and often unnecessary economic costs. The COgnitive behavioural therapy versus standardised medical care for adults with Dissociative non-Epileptic Seizures (CODES) trial is an evaluation of a specifically tailored psychological intervention with the aims of reducing seizure frequency and severity and improving psychological well-being in adults with DS. The aim of this paper is to report in detail the quantitative and economic analysis plan for the CODES trial, as agreed by the trial steering committee.

**Methods:**

The CODES trial is a multicentre, pragmatic, parallel group, randomised controlled trial performed to evaluate the clinical effectiveness and cost-effectiveness of 13 sessions of cognitive behavioural therapy (CBT) plus standardised medical care (SMC) compared with SMC alone for adult outpatients with DS.

**Discussion:**

The objectives and design of the trial are summarised, and the aims and procedures of the planned analyses are illustrated. The proposed analysis plan addresses statistical considerations such as maintaining blinding, monitoring adherence with the protocol, describing aspects of treatment and dealing with missing data. The formal analysis approach for the primary and secondary outcomes is described, as are the descriptive statistics that will be reported. This paper provides transparency to the planned inferential analyses for the CODES trial prior to the extraction of outcome data. It also provides an update to the previously published trial protocol and guidance to those conducting similar trials.

**Trial registration:**

ISRCTN registry ISRCTN05681227 (registered on 5 March 2014); ClinicalTrials.gov NCT02325544 (registered on 15 December 2014).

## Background

### CODES trial

The COgnitive behavioural therapy versus standardised medical care for adults with Dissociative non-Epileptic Seizures (CODES) trial is a multicentre, pragmatic, parallel group, randomised controlled trial (RCT) performed to evaluate the clinical effectiveness and cost-effectiveness of 13 sessions of cognitive behavioural therapy (CBT) plus standardised medical care (SMC) compared with SMC alone in reducing dissociative seizure (DS) frequency and severity and improving seizure freedom, quality of life, psychosocial well-being and cost-effectiveness in terms of health service use in adults with non-epileptic DSs [1]. The protocol paper for the CODES trial has been published previously [1]; the aim of this paper is to report in detail the quantitative and health economic analysis plan as agreed by the trial steering committee in April 2016.

### Dissociative seizures

Approximately 12–20% of patients seen in epilepsy clinics may be having DSs rather than epileptic seizures [2], and rates of DS incidence have been estimated at around 4.9/100,000/year [3]. Although DSs are paroxysmal events that resemble epileptic seizures or syncopes, they are not associated with ictal electroencephalographic discharges. Other common names for DS include *psychogenic non-epileptic seizures* and *non-epileptic attacks*, and they are found under the umbrella headings of dissociative and conversion disorders in psychiatric classifications. DS presents a challenge for clinicians in terms of diagnosis and management. Patients with DS demonstrate high rates of psychiatric comorbidities such as anxiety, depression and post-traumatic stress disorder [4], and it has been shown that they have a raised risk of mortality unrelated to their seizures [5]. Patients with DS may go through expensive or unnecessary interventions; they can sustain injuries during a seizure; and their quality of life is lower than in patients with epilepsy [6]. It has been shown that medical service use and costs can be reduced if a correct diagnosis is given [7].

Despite limited evidence to date for its effectiveness [8–12], psychotherapy is viewed as the treatment of choice for DS [13]; however, the involvement of psychiatrists and psychologists is variable. The National Institute for Health and Care Excellence [14] and the Scottish Intercollegiate Guidelines Network [15] have recognised the need for psychiatric and psychological input for patients with DS, who would benefit from the development of neuropsychiatric care pathways [16] (i.e., bridging the gap between neurology and psychiatry). However, there is little basis on which to recommend a particular type of psychotherapy for this patient group, and care provision in the United Kingdom remains extremely variable.

The CODES trial will therefore permit evaluation of the clinical effectiveness and cost-effectiveness of CBT specifically adapted for patients with DS within a structured care pathway involving neurology, liaison/neuropsychiatry and psychotherapy and should then provide a model for future services and more rational commissioning of care for this patient group. It will provide a basis for the wider training of therapists to work with patients with DS and support the role of psychiatrists in treating this group of patients, who commonly have complex mental health care needs.

### Research objectives

#### Primary objective

Our primary objective is to evaluate the effectiveness of CBT (plus SMC) compared with SMC alone in reducing monthly DS frequency at 12 months post-randomisation.

#### Secondary objectives

Our secondary objectives are to evaluate the effectiveness of the intervention in terms of further secondary outcomes, specifically to assess the following:

1. Reductions in subjective DS severity and disability, as well as improvements in seizure freedom, psychosocial and psychological well-being, and health-related quality of life following CBT plus SMC compared with SMC alone at 12 months post-randomisation
2. A reduction in health service use at 12 months post-randomisation following CBT plus SMC compared with SMC alone
3. The cost-effectiveness of CBT plus SMC compared with SMC alone at 12 months post-randomisation

In addition, we seek to characterise the following:

4. The global clinical improvement shown by patients as a result of either treatment
5. Participants’ satisfaction with either treatment

### Outcome measures

The primary outcome measure is monthly DS frequency at 12 months post-randomisation, defined as seizure occurrence over the previous 4 weeks [1]. This will also be collected at 6 months post-randomisation as an auxiliary variable (*see explanation below*). Seizure frequency data will be recorded by patients in daily seizure diaries and will be collected by the research workers every 2 weeks throughout the trial by whichever means patients find acceptable (diaries, text/phone/online). The research workers will then enter the data as weekly seizure counts into the database system (MACRO; InferMed Ltd, London, UK) set up for this trial by King’s Clinical Trials Unit (KCTU) at the Institute of Psychiatry, Psychology and Neuroscience in London.

Monthly DS frequency will be converted into an incidence rate by the trial statistician. The incidence rate is defined as the number of seizures (count) divided by the number of days. The number of days will depend on how many weekly seizure counts are recorded for each participant at the follow-up time points (7, 14, 21 or 28 days). If the seizure diary has not been collected for the relevant 4 weeks at 6 months (weeks 23–26) and 12 months (weeks 49–52) post-randomisation, an allowance of 2 weeks on either side of these time points will be given to calculate monthly seizure frequency.

An overall self-report estimate of DS frequency in the preceding 4 weeks will also be requested from participants at baseline and at the two follow-up time points to help deal with missing diary data. *See* the trial protocol [1] for further details of all outcome measures and assessment timings.

### Trial design

**Fig. 1:**
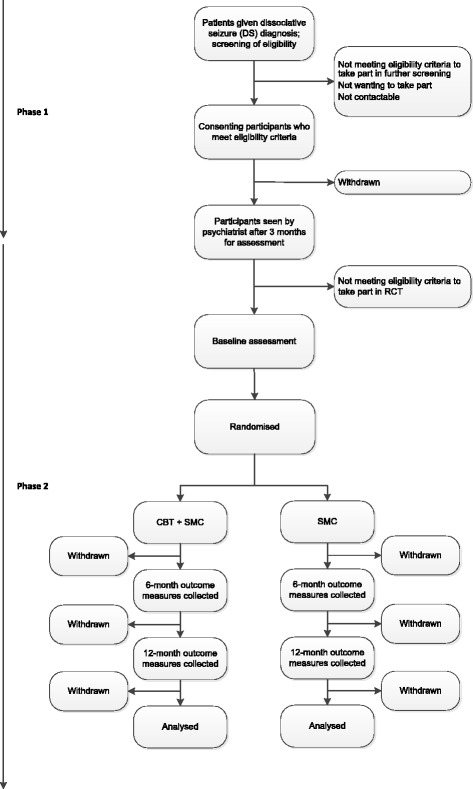
COgnitive behavioural therapy versus standardised medical care for adults with Dissociative non-Epileptic Seizures (CODES) trial Consolidated Standards of Reporting Trials (CONSORT) diagram. *RCT* Randomised controlled trial, *CBT* Cognitive behavioural therapy, *SMC* Standardised medical care

The CODES trial is a multicentre, parallel group, superiority RCT. It comprises a two-stage screening phase: (1) an initial assessment is carried out at recruitment, and (2) a re-assessment is conducted at the psychiatrist visit approximately 3 months later. Further information on this eligibility screening can be found in the protocol publication [1], and an illustration of the screening, recruitment, randomisation and follow-up process is provided in Fig. 1.

Once both phases of consent and baseline assessments are complete, the individuals are randomised to one of the treatment arms. The procedure is as follows. Upon receipt of notification that the patient has consented to participate in the RCT and that the baseline questionnaires have been completed, the local research worker electronically submits details of each participant to the KCTU online randomisation system (www.ctu.co.uk/randomisation). This includes the participant’s identification number, study centre, initials and date of birth. The system generates confirmation emails that can be blinded or unblinded to treatment allocation. Therefore, relevant staff will be notified immediately of the treatment allocation, with or without unblinded details, depending on their role in the study. Specifically, the research workers will receive a blinded confirmation of successful randomisation, and the trial manager and principal investigator will receive an unblinded notification. The CBT therapists delivering the manualised CBT will be informed of the details of the person randomised to that intervention by the trial manager and will liaise with patients to arrange their attendance at appointments.

### Randomisation and blinding

Randomisation is undertaken using a 1:1 ratio and is stratified by liaison/neuropsychiatry centre with variable block sizes within centres to ensure that the distribution of centres is balanced across the two trial arms. There are a total of 18 centres that are involved with patient randomisation.

Blinding is planned for outcome assessors (research workers) and the trial statistician. Evidence for unblinding of treatment to assessors will be studied to ascertain whether they can tell to which treatment arm the participants are randomly allocated for the trial; at 12 months or at withdrawal, the assessors will guess the arm to which they think the participants were allocated. The trial statistician will then compare whether the proportions who guess CBT in each arm is different.

### Sample size

Data are derived from our pilot RCT study (comparing CBT and SMC on a comparable population), which was the largest study at the time informed our sample size calculations [11]. Analysis of seizure outcome in that study was controlled for pre-randomisation seizure frequency, and it revealed a large standardised effect size of Cohen’s *d* = 0.75 [17] (logarithmic scale) for seizure frequency reduction following CBT compared with SMC at the end of CBT treatment (measured at a comparable time point for the SMC group). Of relevance for the calculation of the CODES sample size, our earlier study also yielded a moderate effect size of Cohen’s *d* = 0.42 (logarithmic scale) 6 months after the end of treatment, which broadly approximates the 12-month post-randomisation follow-up point in the CODES trial. We therefore based our sample size estimation on this moderate effect size, which we considered to be clinically meaningful.

We were also able to consider effect sizes for other non-seizure-related outcomes. We found that in other studies of CBT-based psychotherapy for functional symptoms, it is not uncommon to obtain moderate effect sizes. For example, researchers in a large RCT studying patients with chronic fatigue syndrome that permitted a comparison between CBT and standard medical care reported a standardised effect size of *d* > 0.5 at 52 weeks post-randomisation [18]. An RCT study of a brief guided self-help CBT approach for patients with a mixture of functional neurological symptoms (10% of whom had DS) yielded an effect size of *d* = 0.48 at 3 months [19]. Thus, we are aiming to be able to detect an effect size comparable to that found in other CBT-based interventions with patients with functionally/medically unexplained symptoms.

An initial calculation suggested that 121 participants per group were needed to detect an effect size of *d* = 0.42 with 90% power at the 5% significance level using a two-sided *t* test for logarithmic frequencies. However, this number needed to be inflated to allow for therapist effects within the CBT group. Therefore, by using an intra-class correlation coefficient (ICC) of 0.02, which is based on a typical therapist ICC [20], and assuming that around 15 therapists will be delivering CBT, the sample size increased to 149 participants per arm, which would achieve 92.6% power (using the cluspower command in Stata, allowing for clustering in only one trial arm).

As explained further in the Inferential analysis section below, pre-randomisation seizure frequency will be recorded for all of the participants and included as a covariate in the analytical model. This means that the precision of future intervention effect estimates will increase, and so, to account for this, we applied a deflation factor of 0.83, which is based on a correlation between pre-randomisation and follow-up in frequencies of *r* = 0.42 [21]. Finally, attrition needed to be accounted for to re-inflate the sample size. In the pilot RCT, 11% of patients were lost to follow-up [11], but we allowed for a more conservative attrition rate of 17% at 12 months. Therefore, we aim for a final sample size of 149 participants per arm and a total of 298 participants.

### Treatment duration and timing of outcome assessments

CBT therapists who have undergone further specific training for the CODES trial will deliver 12 sessions of CBT plus 1 booster session. CBT will take place over 4–5 months along with a further booster session approximately 9 months after randomisation. All participants will complete follow-up measures at two time points: 6 and 12 months post-randomisation. The aim is for follow-up assessments to be collected up to 4 weeks before and up to 8 weeks after the two time points, where outcomes can be considered reasonably constant. If a large proportion of outcome data is collected outside this stability window, then sensitivity analysis will be considered to adjust for the time difference. Participants randomised to SMC alone will be referred for psychotherapy if they are deemed clinically to require this at the end of the study and after follow-up is complete, but no further data will then be collected on these people, and this treatment course will not form part of the clinical trial.

### Auxiliary information

As explained above, the primary outcome is seizure frequency at 12 months post-randomisation as recorded in patient seizure diaries. We also collect further reports of seizure frequency: seizure frequency at 6 months post-randomisation and patient self-reported number of seizures in the last 4 weeks. We will use such auxiliary information to assess the size of measurement error in our primary outcome and also to predict missing values in the primary outcome analysis (*see below* for description of multiple imputation [MI] process). Regarding measurement precision, Cohen’s kappa statistic and Spearman’s rank correlation coefficients will be calculated to assess inter-measurement method reliability and the strength of any monotonic relationship.

### Data analysis plan

#### Baseline comparability of randomised groups

Baseline descriptions of participants by treatment and overall will be provided as minimums and maximums, means and SDs, and medians and quartiles for continuous variables as appropriate. Frequencies and proportions will be presented for categorical variables. No significance testing will be used to test baseline differences between the trial arms. All baseline variables will be reported overall and by trial arm. These will be grouped into participant demographics and participant clinical information. The primary and secondary outcomes will also be summarised overall and by trial arm.

### Adherence to allocated treatment

Within the CBT arm, adherence to allocated treatment will be described using study-specific therapy logs completed by the CBT therapists. These logs will be used to record attendance at CBT sessions or reasons for non-attendance, as well as rate completion of homework tasks, implementation of activities negotiated in sessions, and whether DSs experienced in session disrupted therapy. Compliance (adherence) is defined as having attended at least nine of the sessions of CBT. A summary of the number of CBT sessions attended and the number of patients compliant with CBT sessions will be provided. Compliant versus non-compliant participants will be compared on baseline variables, and the reasons for withdrawal from treatment will be summarised.

In addition, CBT sessions will be recorded with participants’ consent, and a random selection of these audio recordings will be used to rate therapy integrity to assess the extent to which therapists adhered to the study-specific CBT [1]. The number of participants in the SMC arm who received the active treatment component (CBT) (i.e., treatment contamination) will be described. This will be compared against the health service use data in the economic analysis for consistency.

### Loss to follow-up and other missing data

Withdrawal from trial follow-up (attrition rate) will be reported by intervention group. The proportions of participants with missing values for each variable will be summarised in each arm and at each time point. The reasons for withdrawal from the trial will be summarised.

The baseline characteristics of those missing 12-month follow-up data will be compared with those with complete follow-up. The relationship between baseline characteristics and missing data will also be investigated graphically. Imputation by chained equations (ICE), a form of MI, will be used in the formal analysis to deal with missingness, and predictive baseline characteristics will be included in this process.

The relationship between drop-out from therapy and loss to follow-up will also be described. This will be performed by an independent statistician to maintain blinding of the trial statistician. Binary variables will be constructed for completion of at least nine CBT sessions (“compliant”) and for drop-out at 12 months. The relationship between these two post-randomisation variables within the CBT arm will be assessed by using a chi-square test and will inform the decision whether to include this ‘compliance’ variable in the ICE step, which is explained further in the ‘Inferential analysis’ section below.

### Adverse event reporting

Adverse events, adverse reactions, serious adverse events and serious adverse reactions will be summarised by trial arm and overall.

### Descriptive statistics for outcome measures

Each of the primary and secondary outcome measures as listed in the protocol [1] will be described by treatment group and time point. Means and SDs or medians and IQRs will be used for continuous variables, where relevant. We will check whether continuous outcomes can be assumed to be normally distributed using visual diagnostics, such as residual plots. Histograms and goodness-of-fit tests will be used to assess whether the count variables have a Poisson distribution (i.e., whether the variances equal the means or if there is evidence of over-dispersion). Frequencies and proportions will be used to describe categorical variables.

### Inferential analysis

#### Aims of formal inferences

An intention-to-treat approach will be employed to estimate effectiveness. The formal statistical analyses will estimate the differences in relevant summaries (means, incidence rates) between patients randomised to CBT plus SMC and SMC alone at the various post-treatment observation time points. Group difference estimates and associated 95% CIs will be reported. The significance level will be 5% (two-sided) for the primary outcome. Missing post-randomisation assessments will be dealt with by using the ICE approach for MI. Provided that predictors of missingness are included in the ICE step, the analysis should remain valid in the presence of missing data under the missing at random (MAR) assumption.

The trial statistician will remain blinded until the main analyses have been completed. Any analyses that cannot be performed blinded (e.g., modelling therapist effects in the CBT arm) will be done at the end of the analysis to preserve blinding for as long as possible. Sensitivity analyses will be used to assess the robustness of conclusions to non-ignorable missing outcome data and to departures from randomised treatment.

### Analysis of the primary outcome

The analysis population will include all patients who are randomised. The primary outcome is monthly seizure frequency at 12 months post-randomisation, defined as seizure occurrence over the previous 4 weeks. For the purpose of the primary outcome analysis, this will be taken as measured by the seizure diaries. Other seizure variables will be included in the imputation step, as explained below.

Seizure frequency will be captured by a seizure count and an exposure period. An individual’s incidence rate is then defined by the number of seizures divided by number of days (7, 14, 21 or 28 days), as explained above. The frequency outcome will be modelled using generalised linear mixed models (GLMMs) for Poisson data. The count outcome at 12 months will constitute the dependent variable, and an offset will be used to acknowledge any variability in exposure periods. A Poisson model expresses the effect of the intervention in the form of an incidence rate ratio contrasting the expected number of seizures under CBT plus SMC with the expected number under SMC alone.

The explanatory variables will be seizure frequency at baseline, randomisation stratifier (liaison/neuropsychiatry clinics) and trial arm. The model also contains participant-varying random intercepts to account for any over-dispersion. Potential clustering will be assessed in the model by considering including doctor-varying random intercepts to account for effects of the doctor delivering SMC and therapist-varying intercepts in the CBT arm to account for therapist effects. Poisson model assumption checks are described below.

The model will be estimated using MI (specifically ICE) and will allow for missing outcome data under the MAR assumption. The analysis is valid provided this assumption holds; this means that the observed variables driving missingness have been included in the ICE step. Predictors of missingness (i.e., baseline characteristics and compliance with treatment in the CBT arm) will be included in the ICE step; this is aimed at ensuring that our MAR assumption is realistic.

In addition, all of the explanatory variables used in the analytical model will be included in the ICE step to ensure that the imputation model is at least as general as the analytical model. Importantly, the ICE step will also contain seizure frequency at 6 months and auxiliary variables for seizure outcomes to make a more realistic MAR assumption and gain precision. The effect of departures from this MAR assumption on results will be assessed using sensitivity analyses [22].

### Analysis of secondary outcomes

Secondary patient outcomes relating to DS severity and disability, health-related quality of life, well-being, global clinical improvement and satisfaction with treatment will be analysed using similar GLMMs. For example, continuous variables such as quality of life will be modelled using regression with random effects, accounting for doctor or therapist clustering if necessary. Transformations will be investigated for secondary outcomes that are unlikely to be normally distributed. Binary variables such as seizure freedom in last 3 months (yes/no) will be analysed using logistic regression with random effects. Similarly to the primary outcome analysis, secondary outcome measures at 6 months post-randomisation will be used as auxiliary variables in the imputation model to account for missingness. Secondary outcomes relating to the economic objectives are explained below in the health economic plan.

### Statistical considerations

#### Missing items in scales and subscales

The number (percent) with complete data will be reported. The ideal approach would be to use missing value guidance provided for scales. Where this is not available, scales will be prorated for an individual if 20% of items or less are missing. For example, in a scale with ten items, prorating will be applied to individuals with one or two items missing. The average value for the eight or nine complete items will be calculated for that individual and used to replace the missing values. The scale score will be calculated on the basis of complete values and these replacements.

#### Missing baseline data

We do not anticipate missing values in pre-randomisation variables. However, if we encounter missing baseline values, then we can also use the MI process as explained above, or they can be singly imputed according to the method of White and Thompson [23].

### Method for handling multiple comparisons

There is only a single primary outcome, and no formal adjustment of *p* values for multiple testing will be applied. However, care should be taken when interpreting the numerous secondary outcomes.

### Method for handling non-compliance

In addition to the primary intention-to-treat analysis, the effect of actually receiving treatment as defined in the protocol will also be estimated. If non-compliance with treatment is high in the CBT arm, a complier average causal effect (CACE) analysis will be considered. CACE analysis examines the effect of CBT among compliers. Complier status is observable only in the CBT arm. Instrumental variable methods using randomisation as an instrument for CBT receipt will enable estimation of CACE without incurring bias [24].

### Model assumption checks

To assess the adequacy of the Poisson regression model for the primary outcome, we will first look at the basic descriptive statistics for the count data. A Poisson model assumes that each seizure is independent of each other and that the count mean and variance are similar; therefore, if they are very different, this may be an issue of over-dispersion. A goodness-of-fit chi-square test will be performed to assess the model fit; if the test is not statistically significant, then the Poisson model fits well, and distributional assumptions are met. However, if the Poisson assumptions are violated, then a negative binomial model may be considered more appropriate because an extra parameter can model the over-dispersion. For the secondary outcomes, regression residuals will be plotted to check for normality and outliers.

### Subgroup analyses

No subgroup analyses are planned. The study is not powered to investigate interaction effects. In addition, this analysis plan does not cover any further secondary analyses of the trial dataset. Mediator and exploratory moderator analyses may be performed after the primary trial data analysis.

### Software

#### Data management

An online data collection system for clinical trials (MACRO) will be used. This is hosted on a dedicated server at King’s College London and managed by the KCTU.

#### Statistical analysis

Stata version 14.0 software (StataCorp, College Station, TX, USA) will be used for data description and inferential analysis.

### Economic analysis plan

#### Health economic objective and measures

We will take both a health/social care and a societal perspective in the assessment of cost-effectiveness. Societal costs include lost employment and care from family/friends. Permission to use Hospital Episode Statistics data will be applied for to measure inpatient and other hospital use; however, if this is not possible, then service use will still be measured with the Client Service Receipt Inventory [25] questionnaire, which is collected at baseline and at 6- and 12-month follow-up, and this will supplement information on number of therapy sessions provided.

Costs will be calculated by combining the service use data with recognised unit costs [26, 27]. Wage rates will be used to value lost work and care from family/friends. Intervention unit costs will be based on salaries, overhead costs, training and supervision.

Costs will be compared between the two arms at 6- and 12-month follow-up (the latter being the cumulative costs over the entire follow-up period). Baseline costs will be controlled for in a bootstrapped regression model (given the likely skewed data distribution). We will report 95% CIs around the cost difference at each time point.

To assess cost-effectiveness, costs will be combined with change in DS frequency and also quality-adjusted life-years (QALYs) derived from the five-level European Quality of Life-5 Dimensions (EQ-5D-5L) [28] using the AUC approach. Incremental costs and outcomes will be obtained via regression models, and 1000 differences obtained from bootstrapped resamples will be plotted on a cost-effectiveness plane to investigate uncertainty around the incremental cost-effectiveness ratio obtained from point estimates.

Sensitivity analyses will be conducted with missing follow-up costs and QALYs derived via MI. If an individual has a missing number of service contacts or a missing EQ-5D domain score, then the median of others with these data will be used. Other sensitivity analyses will use QALYs derived via the SF-6D, derived from the 12-item Medical Outcomes Study Short Form Health Survey version 2.0 (SF-12v2) [29, 30]. Furthermore, we will use home care workers as alternative values for informal care. There has been limited previous research in this area, and this trial will provide evidence on the impact of CBT in patients with DS over a 1-year follow-up.

### Trial status

Recruitment completed on May 31st 2017 and the trial is now in final follow-up stages.

## Abbreviations

CACE: Complier average causal effect
CBT: Cognitive behavioural therapy
CODES: COgnitive behavioural therapy versus standardised medical care for adults with Dissociative non-Epileptic Seizures
CONSORT: Consolidated Standards of Reporting Trials
DS: Dissociative seizure
EQ-5D-5L: Five-level European Quality of Life-5 Dimensions
ICC: Intra-class correlation coefficient
GLMM: Generalised linear mixed model
ICE: Imputation by chained equations
KCTU: King’s Clinical Trials Unit
MAR: Missing at random
MI: Multiple imputation
QALY: Quality-adjusted life-year
RCT: Randomised controlled trial
SF-12v2: 12-Item Medical Outcomes Study Short Form Health Survey version 2.0
SMC: Standardised medical care

## References

[1] Goldstein LH, Mellers JD, Landau S, Stone J, Carson A, Medford N (2015). COgnitive behavioural therapy vs standardised medical care for adults with Dissociative non-Epileptic Seizures (CODES): a multicentre randomised controlled trial protocol. BMC Neurol.

[2] Angus-Leppan H (2008). Diagnosing epilepsy in neurology clinics: a prospective study. Seizure.

[3] Duncan R, Razvi S, Mulhern S (2011). Newly presenting psychogenic nonepileptic seizures: incidence, population characteristics, and early outcome from a prospective audit of a first seizure clinic. Epilepsy Behav.

[4] Brown RJ, Reuber M (2016). Psychological and psychiatric aspects of psychogenic non-epileptic seizures (PNES): a systematic review. Clin Psychol Rev.

[5] Duncan R, Oto M, Wainman-Lefley J (2012). Mortality in a cohort of patients with psychogenic non-epileptic seizures. J Neurol Neurosurg Psychiatry.

[6] Al Marzooqi SM, Baker GA, Reilly J, Salmon P (2004). The perceived health status of people with psychologically derived non-epileptic attack disorder and epilepsy: a comparative study. Seizure.

[7] Razvi S, Mulhern S, Duncan R (2012). Newly diagnosed psychogenic nonepileptic seizures: health care demand prior to and following diagnosis at a first seizure clinic. Epilepsy Behav.

[8] Goldstein LH, Mellers JDC (2012). Recent developments in our understanding of the semiology and treatment of psychogenic nonepileptic seizures. Curr Neurol Neurosci Rep.

[9] Reuber M, Mayor R (2012). Recent progress in the understanding and treatment of nonepileptic seizures. Curr Opin Psychiatry.

[10] Martlew J, Pulman J, Marson AG (2014). Psychological and behavioural treatments for adults with non-epileptic attack disorder. Cochrane Database Syst Rev.

[11] Goldstein LH, Chalder T, Chigwedere C, Khondoker MR, Moriarty J, Toone BK (2010). Cognitive-behavioral therapy for psychogenic nonepileptic seizures: a pilot RCT. Neurology.

[12] LaFrance WC, Baird GL, Barry JJ, Blum AS, Frank Webb A, Keitner GI (2014). Multicenter pilot treatment trial for psychogenic nonepileptic seizures: a randomized clinical trial. JAMA Psychiatry.

[13] Mayor R, Smith PE, Reuber M (2011). Management of patients with nonepileptic attack disorder in the United Kingdom: a survey of health care professionals. Epilepsy Behav.

[14] National Institute for Health and Care Excellence (NICE) (2004). The epilepsies: the diagnosis and management of the epilepsies in adults and children in primary and secondary care. NICE Clinical Guideline CG20.

[15] Scottish Intercollegiate Guidelines Network (SIGN) (2005). Diagnosis and management of epilepsy in adults. SIGN Guidelines 70.

[16] Agrawal N, Fleminger S, Ring H, Deb S (2008). Neuropsychiatry in the UK: national survey of existing service provision. Psychiatr Bull.

[17] Cohen J (1988). Statistical power analysis for the behavioral sciences.

[18] White PD, Goldsmith KA, Johnson AL, Potts L, Walwyn R, DeCesare JC (2011). Comparison of adaptive pacing therapy, cognitive behaviour therapy, graded exercise therapy, and specialist medical care for chronic fatigue syndrome (PACE): a randomised trial. Lancet.

[19] Sharpe M, Walker J, Williams C, Stone J, Cavanagh J, Murray G (2011). Guided self-help for functional (psychogenic) symptoms: a randomized controlled efficacy trial. Neurology.

[20] Baldwin SA, Murray DM, Shadish WR, Pals SL, Holland JM, Abramowitz JS (2011). Intraclass correlation associated with therapists: estimates and applications in planning psychotherapy research. Cogn Behav Ther.

[21] Borm GF, Fransen J, Lemmens WA (2007). A simple sample size formula for analysis of covariance in randomized clinical trials. J Clin Epidemiol.

[22] White IR, Horton NJ, Carpenter J, Pocock SJ (2011). Strategy for intention to treat analysis in randomised trials with missing outcome data. BMJ.

[23] White IR, Thompson SG (2005). Adjusting for partially missing baseline measurements in randomized trials. Stat Med.

[24] Dunn G, Maracy M, Dowrick C, Ayuso-Mateos JL, Dalgard OS, Page H (2003). Estimating psychological treatment effects from a randomised controlled trial with both non-compliance and loss to follow-up. Br J Psychiatry.

[25] Beecham J, Knapp M. Costing psychiatric interventions. In: Thornicroft G, editor. Measuring mental health needs. 2nd ed. London: Gaskell; 2001. p. 200-24.

[26] Curtis L (2011). Unit costs of health and social care 2011.

[27] Department of Health (2012). NHS reference costs.

[28] Herdman M, Gudex C, Lloyd A, Janssen M, Kind P, Parkin D (2011). Development and preliminary testing of the new five-level version of EQ-5D (EQ-5D-5L). Qual Life Res.

[29] Ware J, Kosinksi M, Keller SD (1996). A 12-item short-form health survey: construction of scales and preliminary tests of reliability and validity. Med Care.

[30] Brazier JE, Roberts J (2004). The estimation of a preference-based measure of health from the SF-12. Med Care.

